# Theory of Hot-Carrier Generation in Bimetallic Plasmonic Catalysts

**DOI:** 10.1021/acsphotonics.3c00715

**Published:** 2023-09-15

**Authors:** Hanwen Jin, Matias Herran, Emiliano Cortés, Johannes Lischner

**Affiliations:** † Department of Materials, Imperial College London, South Kensington Campus, London SW7 2AZ, United Kingdom; ‡ Nanoinstitute Munich Faculty of Physics, Ludwigs-Maximilians-Universität München, 80539 Munich, Germany; § Department of Materials and the Thomas Young Centre for Theory and Simulation of Materials, Imperial College London, South Kensington Campus, London SW7 2AZ, United Kingdom

**Keywords:** plasmonics, hot carriers, photocatalysis, bimetallic, modeling

## Abstract

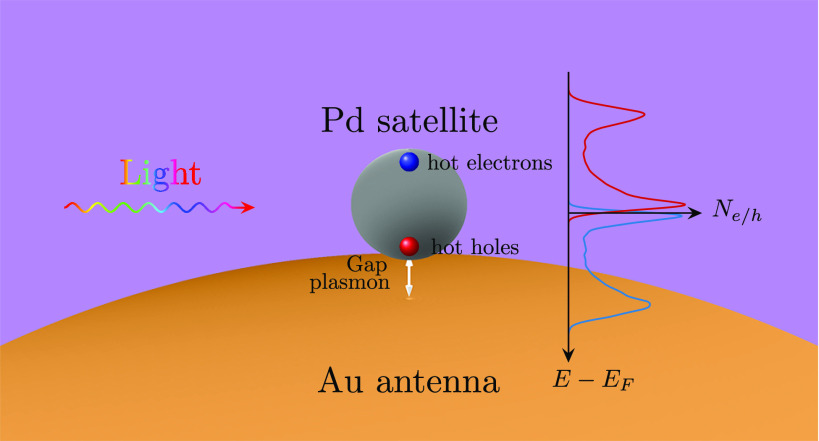

Bimetallic nanoreactors in which a plasmonic metal is used to funnel solar energy toward a catalytic metal have recently been studied experimentally, but a detailed theoretical understanding of these systems is lacking. Here, we present theoretical results of hot-carrier generation rates of different Au–Pd nanoarchitectures. In particular, we study spherical core–shell nanoparticles with a Au core and a Pd shell as well as antenna–reactor systems consisting of a large Au nanoparticle that acts as an antenna and a smaller Pd satellite nanoparticle separated by a gap. In addition, we investigate an antenna–reactor system in which the satellite is a core–shell nanoparticle. Hot-carrier generation rates are obtained from an atomistic quantum-mechanical modeling technique which combines a solution of Maxwell’s equation with a tight-binding description of the nanoparticle electronic structure. We find that antenna–reactor systems exhibit significantly higher hot-carrier generation rates in the catalytic material than the core–shell system as a result of strong electric field enhancements associated with the gap between the antenna and the satellite. For these systems, we also study the dependence of the hot-carrier generation rate on the size of the gap, the radius of the antenna nanoparticle, and the direction of light polarization. Overall, we find a strong correlation between the calculated hot-carrier generation rates and the experimentally measured chemical activity for the different Au–Pd photocatalysts. Our insights pave the way toward a microscopic understanding of hot-carrier generation in heterogeneous nanostructures for photocatalysis and other energy-conversion applications.

## Introduction

There is currently significant interest in harnessing energetic or “hot” electrons and holes generated in metallic nanoparticles for applications in photocatalysis,^1−5^ photovoltaics,^6−8^ and sensing.^10−17^ Metallic nanoparticles feature localized surface plasmons (LSPs) that give rise to large light absorption cross sections.^18^ The LSP has a short lifetime (typically on the order of tens of femtoseconds). Among the various decay mechanisms, the Landau damping decay plays a prominent role because it results in the generation of hot carriers.^19,20^

However, photocatalytic hot-carrier devices often have relatively low efficiencies.^21^ A possible explanation for this is that standard plasmonic materials, such as Au and Ag, are generally not good catalysts.^22^ Therefore, attempts have been made to combine plasmonic materials with catalytic materials, such as Pt, Pd, or Rh, into functional nanoarchitectures. Examples of such heterostructures include Janus nanoparticles,^9^ core–shell systems,^23,24^ or nanoparticle dimers and trimers.^25^ Recently, Herran and co-workers studied different nanoarchitectures of Pd and Au, including core–shell nanoparticles and antenna–reactor systems in which a large Au nanoparticle is “decorated” with small Pd nanoparticles (or satellites), for the production of H_2_ from formic acid.^26^ These authors found significant enhancements in H_2_ production upon illumination of the plasmocatalyst with the largest increase in chemical activity for antenna–reactor systems. Despite these advances, however, there is still no detailed microscopic understanding of the catalytic activity in bimetallic nanoarchitectures.^27^

Insights into microscopic hot-carrier processes, including their generation, thermalization, and extraction, can be gained from theoretical modeling. Atomistic first-principle techniques, such as ab initio time-dependent density-functional theory, can be used to investigate hot-carrier processes in very small nanostructures,^28^ but are challenging to apply to experimentally relevant system sizes. On the other hand, nonatomistic approaches, such as jellium or spherical well models, can be applied to large systems, but do not capture important aspects, including d-band derived nanoparticle states or facet-specific surface properties.^40−46^ To address this challenge, Jin and co-workers recently developed a new approach that combines a solution of Maxwell’s equation with large-scale atomistic tight-binding models which enables the modeling of hot-carrier processes in nanoparticles containing millions of atoms.^39^ So far, however, this approach has only been applied to spherical nanoparticles.

In this paper, we use the method of Jin and co-workers^39^ to study the enhancement of hot-carrier generation rates in a catalytic metal (Pd) induced by the presence of a plasmonic metal (Au) in different bimetallic plasmo-catalytic nanoarchitectures. In particular, we study Au@Pd core–shell nanoparticles and antenna-reactor systems consisting of a large Au nanoparticle which acts as an antenna and a small satellite nanoparticle. The satellite is either a spherical Pd nanoparticle or a Au@Pd core–shell nanoparticle. We compare our results to the hot-carrier generation rates in spherical Pd nanoparticles. We find that the largest hot-carrier generation rates in the catalytic metal are found in antenna-reactor systems, in particular, in those where the satellite nanoparticle is a core–shell system. This can be explained by the large enhancement of the electric field arising from the strongly confined plasmon mode associated with the gap between the antenna nanoparticle and the satellite nanoparticle. We also explore the dependence of hot-carrier generation rates on the light polarization, the size of the antenna nanoparticle, and the gap size between the antenna nanoparticle and the satellite. We compare our calculated hot-carrier generation rates to experimentally measured H_2_ production rates^26^ for different Au–Pd photocatalysts and find a strong correlation between the two. The insights resulting from our work pave the way toward a microscopic design of heterogeneous nanoarchitectures for energy conversion devices. The approach can be readily applied to other materials.

## Methods

### Hot-Carrier Generation Rates

**Figure 1:**
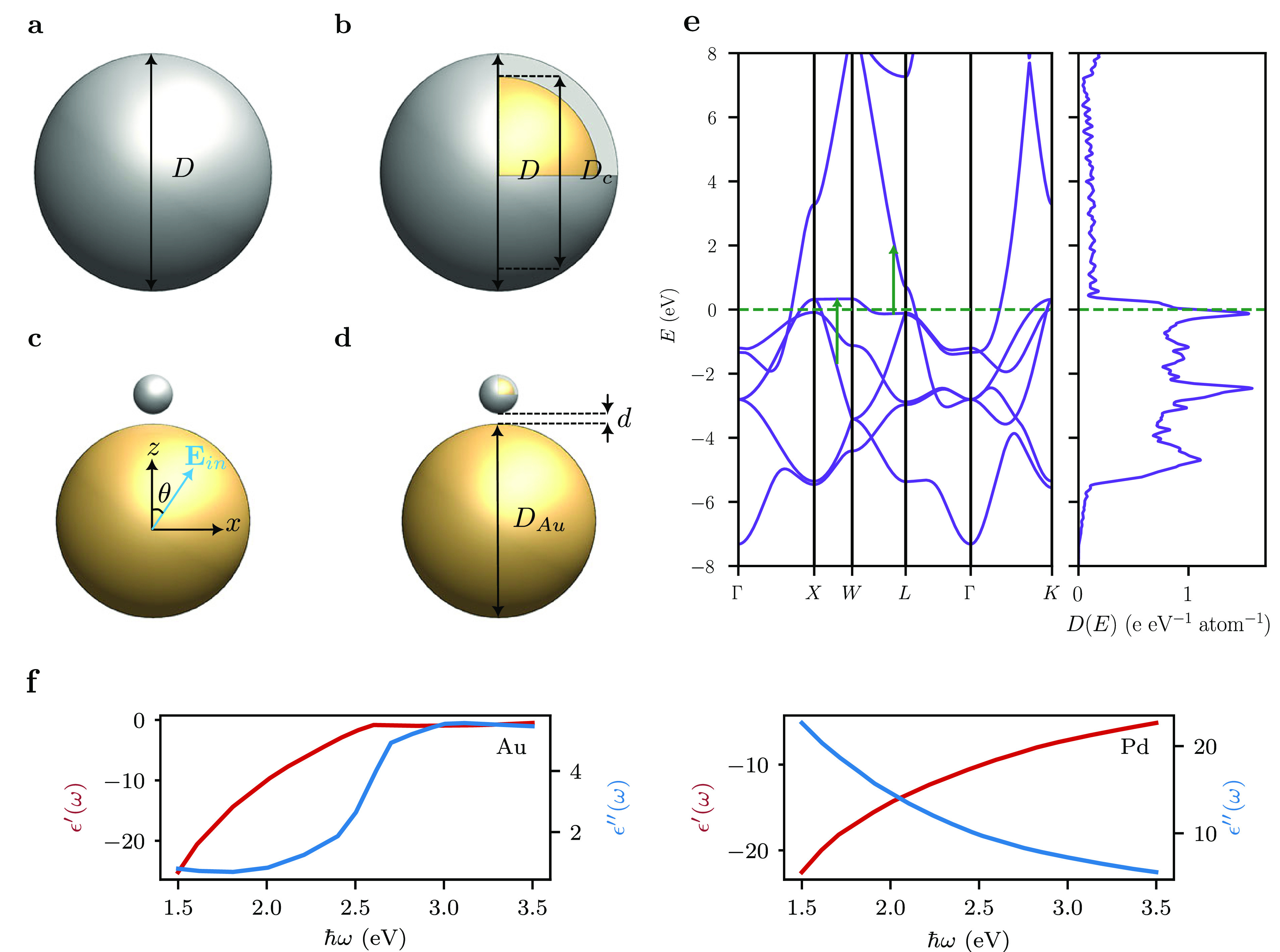
Schematic illustration of (a) a spherical Pd nanoparticle, (b) a Au@Pd core–shell nanoparticle, (c) a Au–Pd antenna–reactor system, and (d) a Au–Au@Pd antenna-reactor system, and (e) band structure of bulk fcc Pd, the zero of energy is set to the Fermi level (indicated by the green dashed line). Green arrows indicate interband transitions involving flat d-bands near the Fermi level. (f) Real and imaginary parts of the dielectric functions of Au and Pd. From ref (48).

We use the approach developed by Jin and co-workers^39^ to calculate hot-carrier generation rates in Pd–Au nanoarchitectures. In this method, the generation rate of hot electrons *N*_*e*_(*E*, ω) of energy *E* excited by light of frequency ω is obtained by evaluating Fermi’s golden rule according to^40^
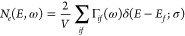
1where *i* and *f* label the initial and final state with energy *E*_*i*_ and *E*_*f*_, respectively. Also, *V* is the volume of the nanoparticle, and we define  with σ = 0.05 eV being standard deviation of the Gaussian which reflects the quasiparticle line width. In the above, Γ_*if*_ is given by
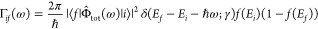
2where *f*(*E*) is the Fermi–Dirac distribution (for a continuous wave photocatalysis experiment, the electron occupation will not necessarily follow a Fermi–Dirac distribution; however, calculating the full nonequilibrium distribution function would require a detailed theory of relaxation effects, which goes beyond the scope of the current manuscript; Fermi’s Golden Rule was evaluated using distributions other than Fermi–Dirac in refs (50 and 51)) with temperature *T* = 298 K, γ = 0.06 eV is a broadening parameter which reflects the line width of an electron–hole pair excitation (here we have used typical values for the broadening parameters σ and γ based on our previous work, in which the effect of electron–electron and electron–phonon interactions are studied in detail;^43^ we have verified that the calculated hot-carrier generation rates are not sensitive to the precise values of the broadening parameters) and  denotes the total potential inside the nanoparticle. This potential is calculated using the quasistatic approximation.^33−38^ In particular, we use the finite element method as implemented in COMSOL^47^ to solve the Laplace equation
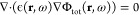
3where ϵ(**r**, ω) is dielectric function of the material at position **r**. We use experimental dielectric functions for Au^48^ and Pd,^48^ see Figure 1f. In the calculations, we first specify the geometry of the nanoarchitecture and the external potential Φ_ext_(**r**, ω) = −**E**_0_·**r** with **E**_0_ denoting the corresponding electric field and then solve Laplace’s equation subject to the far-field condition that
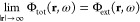
4

Once the total potential of the full Au–Pd nanoarchitecture is determined, we evaluate the hot-carrier generation rate in the Pd subsystem (because only the hot carriers in the Pd are catalytically active). Note that our approach does not capture charge transfer processes between the Au and the Pd which can play an important role in core–shell nanoparticles.^30,31^ In contrast, charge transfer not expected to be important in antenna-satellite system because of the finite gap (caused by the presence of surfactants) between the antenna and the satellite nanoparticle.^26^ The electronic states of the Pd subsystem are obtained using the tight-binding method. For this, we first determine the atomic positions by carving the desired Pd shape (either a spherical nanoparticle or a spherical nanoshell) from the bulk material. The eigenstates of the Hamiltonian are expressed in terms of linear combinations of atomic orbitals according to |*i*⟩ = *∑*_*J*,α_*C*_*J*,α_|*J*, α⟩, with *J* labeling atoms and α labeling orbitals. For each Pd atom, the basis consists of five 4d orbitals, one 5s orbital and three 5p orbitals. The hopping and onsite energies of the Pd tight-binding model are taken from the ”Handbook of the Band Structures of Elemental Solids”.^29^

The matrix element in eq 1 is evaluated using^32^
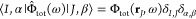
5where **r**_*J*_ denotes the position of atom *J*, and we have ignored the transition dipole contribution to the matrix elements (which we have found to be small in our previous calculations since it does not scale with the nanoparticle size^39^).

The total generation rate of hot carriers (both electrons and holes) per unit volume is given by
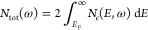
6with *E*_F_ denoting the Fermi energy. The hot hole generation rate *N*_*h*_(*E*, ω) is obtained by replacing *E*_*f*_ with *E*_*i*_ in eq 1.

## Results

We have calculated hot-carrier generation rates in four systems; see Figure 1: (a) spherical Pd nanoparticles consisting of 3589 atoms (corresponding to a diameter *D* = 4.42 nm), (b) spherical Au@Pd core–shell nanoparticles with a Au core (of diameter *D*_c_ = 4.90 nm) and a Pd shell (of thickness 0.40 nm containing 3740 Pd atoms), (c) a Au–Pd antenna–reactor architecture consisting of a small spherical Pd nanoparticle of the same size as in (a), which is separated by a small gap from a larger Au nanoparticle, and (d) a Au–Au@Pd antenna–reactor architecture in which the small nanoparticle has a core–shell structure as in (b). The sizes of the Pd nanoparticle and of the Au@Pd core–shell nanoparticle are similar to the satellite nanoparticles used in the experiment of Herran and co-workers.^26^ Note that we are studying hot-carrier generation only in the Pd subsystems of the various Au–Pd photocatalysts. As all photocatalysts contain a similar number of Pd atoms (and we further report the hot-carrier generation rate per unit volume), we are confident that a quantitative comparison of the hot-carrier generation rates of the different photocatalysts is physically meaningful.

**Figure 2:**
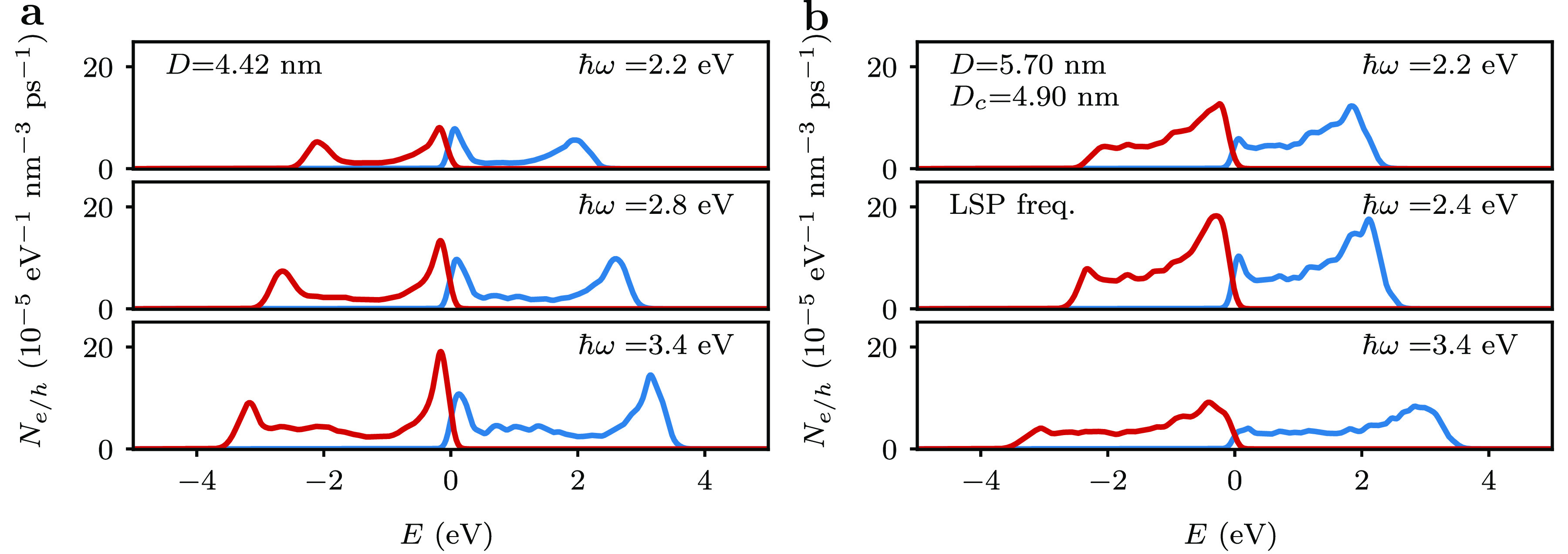
Dependence of hot-carrier generation rate on photon energy for (a) a spherial Pd nanoparticle with a diameter of *D* = 4.42 nm and (b) a Au@Pd core–shell nanoparticle with a core diameter of *D*_c_ = 4.90 nm and a shell thickness of 0.40 nm. Hot hole (electron) generation rates are shown in red (blue). The zero energy is set to the Fermi level.

Figure 2a shows the evolution of the hot carrier generation rate for the spherical Pd nanoparticle as a function of photon energy. Both the hot hole and the hot electron rates exhibit two peaks. The hot hole rate has a peak near the Fermi level, with the hot electron rate having a corresponding peak at ∼*ℏω* above the Fermi level. The other peak of the hot hole rate is at ∼−*ℏω* and the hot electron rate has a corresponding peak just above the Fermi level. As the photon energy is varied, the peaks near the Fermi level are pinned at their positions, but the other peaks move to higher (in the case of the hot electron rate) and lower (in the case of the hot hole rate) energies. This finding can be understood from an analysis of the electronic structure of Pd. In particular, the band structure of Pd, see Figure 1e, exhibits flat d-bands just above and below the Fermi energy. These flat bands give rise to a large density of states, which translates into a high hot carrier generation rate near the Fermi level. The positions of the other peaks are then determined by energy conservation, i.e., for each electron of energy *E*, a corresponding hole of energy *E* – *ℏω* must be created. Finally, we do not observe a dramatic enhancement of the hot-carrier rates at a particular photon energy, which reflects the absence of a strong LSP resonance in spherical Pd nanoparticles.

Figure 2b shows the dependence of the hot carrier generation rate of a Au@Pd core–shell nanoparticle on the photon energy. Again, the rates exhibit peaks near the Fermi level. Some differences in the shapes of the hot-carrier generation rates can be observed compared to the spherical Pd nanoparticle: these are caused by the increase of surface area of the thin Pd shell which enhances the generation of hot carriers from intraband transitions.^39^ In the core–shell system, an enhancement of the hot-carrier generation rate at a photon frequency of 2.4 eV can be observed. This energy is close to the LSP energy of a spherical Au nanoparticle. In other words, at this photon energy the field enhancement of the Au core increases the hot-carrier generation in the Pd shell.^31^

**Figure 3:**
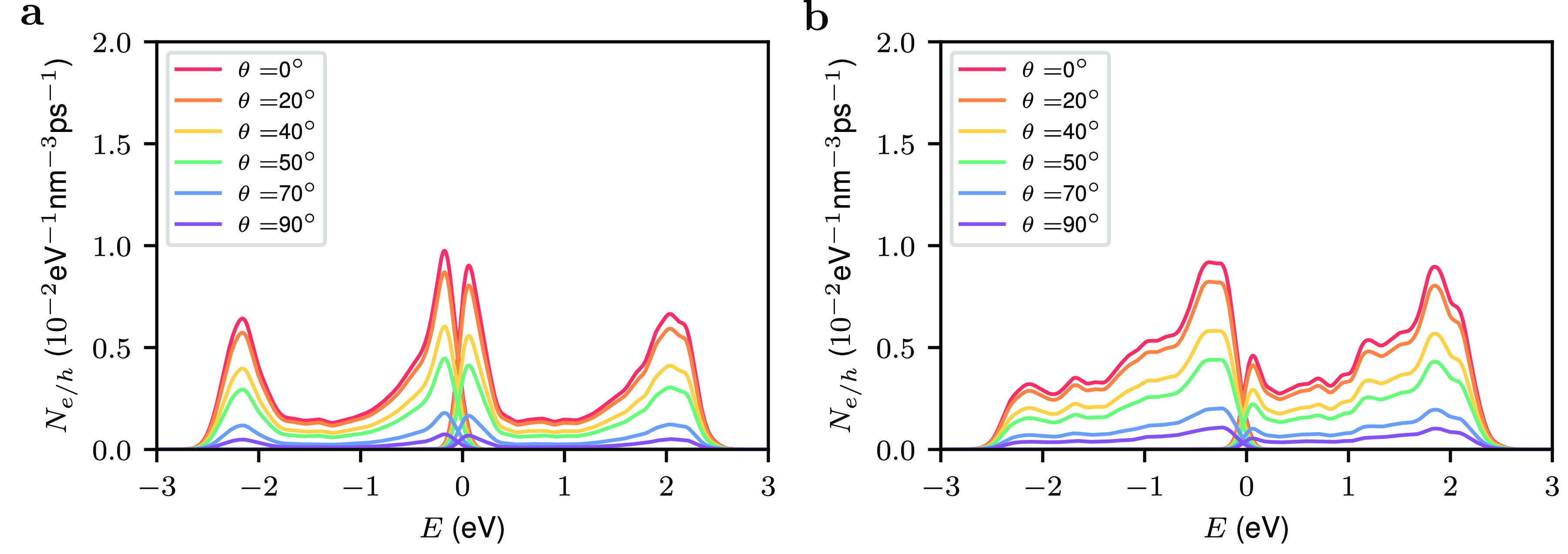
Dependence of hot-carrier generation rate on light polarization for (a) a Au–Pd antenna-reactor system with a diameter of 4.42 nm and (b) Au–Au@Pd reactor system, with a core diameter of 4.9 nm and a total diameter of 5.7 nm. The diameter of the Au nanoparticle is 49 nm. The size of the gap between the Pd and Au nanoparticles is 0.40 nm and the photon energy is 2.28 eV for Au–Pd system and 2.20 for Au–Au@Pd system.

Next, we investigate hot-carrier generation in a Au–Pd antenna–reactor system. Adding the Au nanoparticle to the spherical Pd nanoparticle raises the rotational symmetry of the Pd system. As a consequence, the hot-carrier generation rate now depends on the polarization vector of the electric field. We assume that both the center of the Au and the center of the Pd nanoparticle lie on the *z*-axis, see Figure 1, and describe the electric field through its polar angle θ. Figure 3a shows the dependence of the hot carrier rate on θ. The hot-carrier generation rate is largest when θ = 0° and then decreases as θ is increased. In particular, we find that the hot-carrier generation rate is proportional to cos θ, i.e., *N*_*e*_(ω, *E*, θ) = *N*_*e*_(ω, *E*, 0) cos θ. When θ = 0, the charge carriers in the Au nanoparticle are pushed toward the Pd nanoparticle creating a strongly enhanced electric field. The field is further enhanced by the confinement effect of the small gap between the Au and the Pd nanoparticles giving rise to a so-called gap plasmon.^49^ We note that a large number of satellites is attached to the Au nanoparticles in the experiments of Herran and co-workers.^26^ Also, the light used in experiments is not polarized. Therefore, the experimentally measured hot-carrier generation rate is an average over θ.

Figure 3 shows the same results for a Au–Au@Pd antenna-reactor system. Again, the hot-carrier generation rate of the core–shell system has a shape different from that of the pure Pd system, but it exhibits a similar dependence on the light polarization.

**Figure 4:**
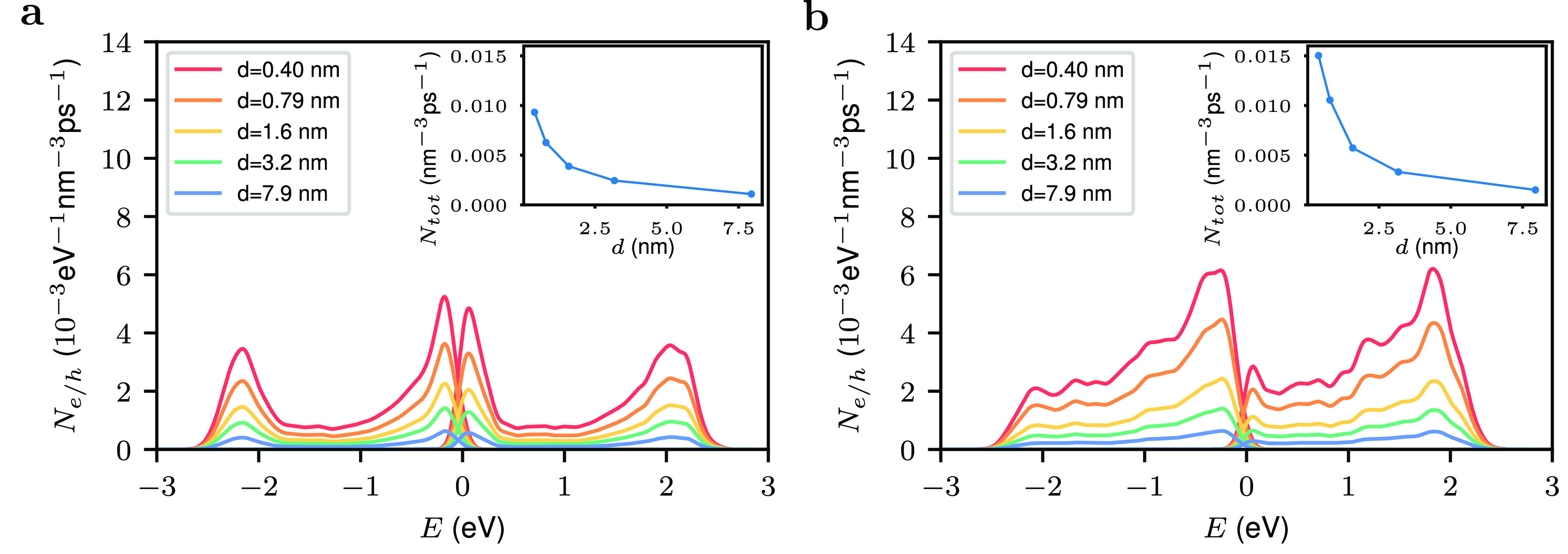
Dependence of the hot-carrier generation of Au–Pd antenna–reactor systems on the size of the gap *d* between the Au nanoparticle and the satellite. (a) Results for a Pd satellite. (b) Results for a Au@Pd core–shell satellite. The diameter of the Au antenna nanoparticle is 49 nm; the photon energy is 2.28 eV for Au–Pd system and 2.20 for Au–Au@Pd system, and the calculation is averaged over all polarization angles. The insets show the total hot-carrier generation rate.

Next, we study the dependence of the hot-carrier generation rate of the two antenna-reactor systems on the size of the gap *d* between the Au nanoparticle and the satellite; see Figure 4. In practice, this parameter is determined by the size of the ligands on the surface of the nanoparticles and is difficult to control experimentally (in the experiment of Herran et al., the gap is estimated to be approximately 1 nm^26^). We find that the largest hot-carrier rates are obtained for the systems with the smallest gaps. The insets show that the total number of hot carriers decreases quickly as the gap size is increased. For example, increasing the gap from 0.49 to 1.6 nm reduces the total number of hot carriers by a factor of 0.38 (Au–Au@Pd) and 0.42 (Au–Pd) and further increase to *d* = 3.2 nm gives rise to an additional reduction by a factor of 0.58 (Au–Au@Pd) and 0.63 (Au–Pd) in the total hot carrier generation rate. This result demonstrates the importance of the confinement effect associated with the gap between the Au and the satellite particles on the strength of the electric field.

**Figure 5:**
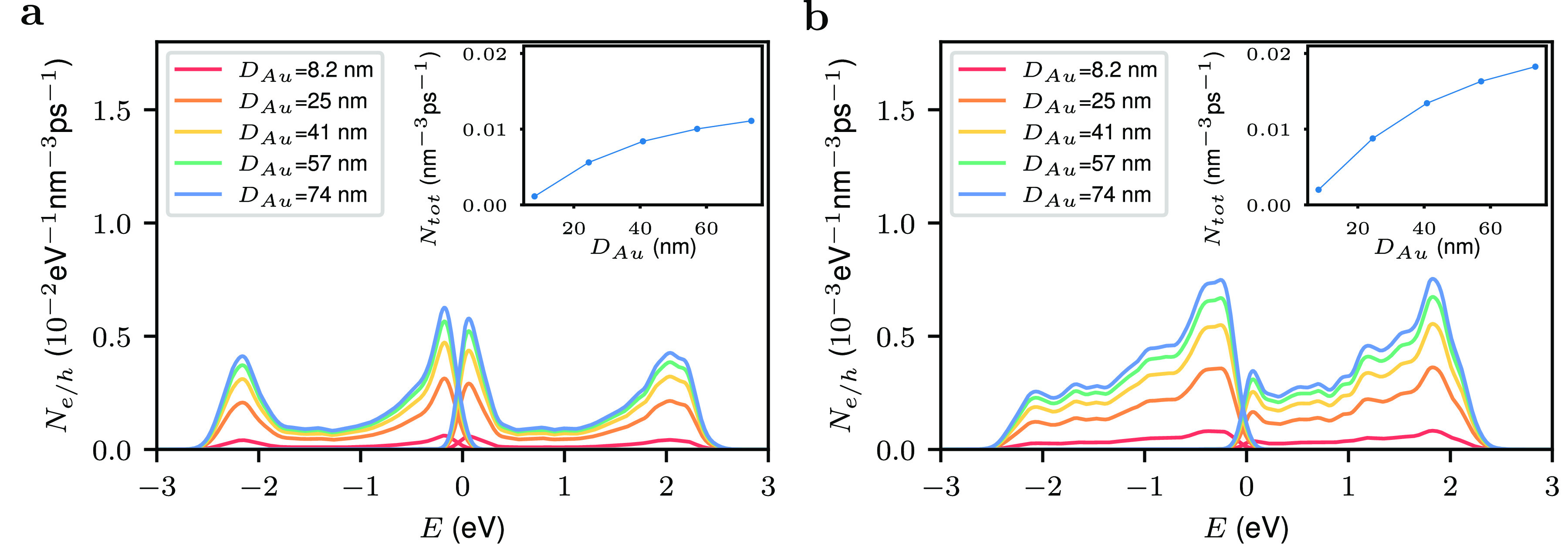
Dependence on the hot-carrier generation of Au–Pd antenna-reactor systems on the size of the Au nanoparticle. (a) Results for a Pd satellite. (b) Results for a Au@Pd core–shell satellite. The size of the gap is 0.40 nm, the photon energy is 2.28 eV for Au–Pd system and 2.20 eV for Au–Au@Pd system, and the calculation was averaged over all polarization angles. The insets show the total hot-carrier generation rates.

Next, we study the dependence of the Pd hot-carrier generation rate of the reactor systems on the size of the Au nanoparticle; see Figure 5. We find that the hot-carrier generation rate increases as the size of the Au nanoparticle increases. Increasing the Au nanoparticle size while keeping the distance between the Au nanoparticle and the satellite fixed reduces the volume available in the gap between the two nanoparticles and thus enhances confinement effects. This in turn leads to electric field enhancement. As the Au nanoparticle size increases, the additional reduction of the gap volume becomes smaller and smaller and only results in a small increase in hot carrier production.

**Figure 6:**
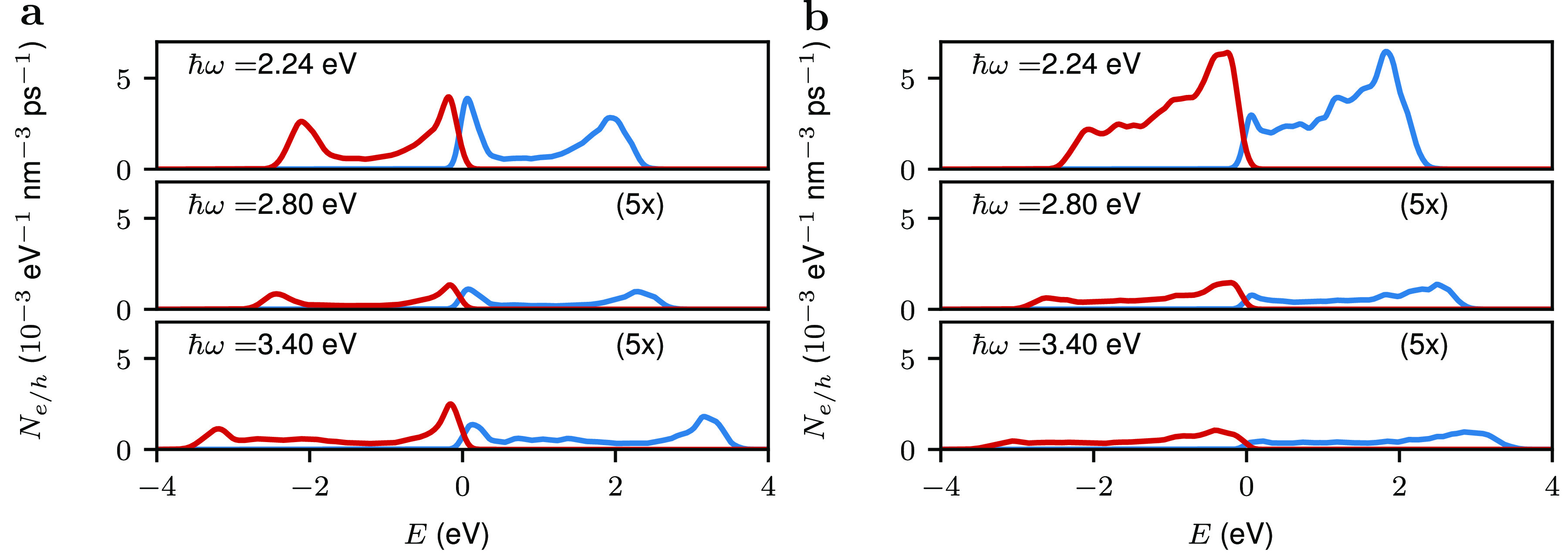
Dependence of hot-carrier generation rate on the photon energy for (a) a Au–Pd antenna–reactor system with a diameter of 4.42 nm and (b) a Au–Au@Pd antenna–reactor system, with a core diameter of 4.9 nm and a total diameter of 5.7 nm. The diameter of the Au nanoparticle is 49 nm. The size of the gap between the Pd and Au nanoparticles is 0.40 nm, and the calculation is averaged over all polarization angles. For visual aid, the hot-carrier generation rates at *ℏω* = 2.80 and 3.40 eV were multiplied by a factor of 5.

Finally, we study the dependence of the hot-carrier generation rates of the reactor systems on the photon energy; see Figure 6. For both systems, a dramatic enhancements of the rates is observed at a photon energy of 2.24 eV compared to the other photon energies. This photon energy is close to the LSP energy of the Au nanoparticle (note that the presence of the satellite modifies the LSP energy of the Au nanoparticle) so the increase of the hot-carrier generation rates reflects the electric field enhancement caused by the plasmon mode. The LSP acts as an optical lens which efficiently funnels energy toward the catalytic material.

**Figure 7:**
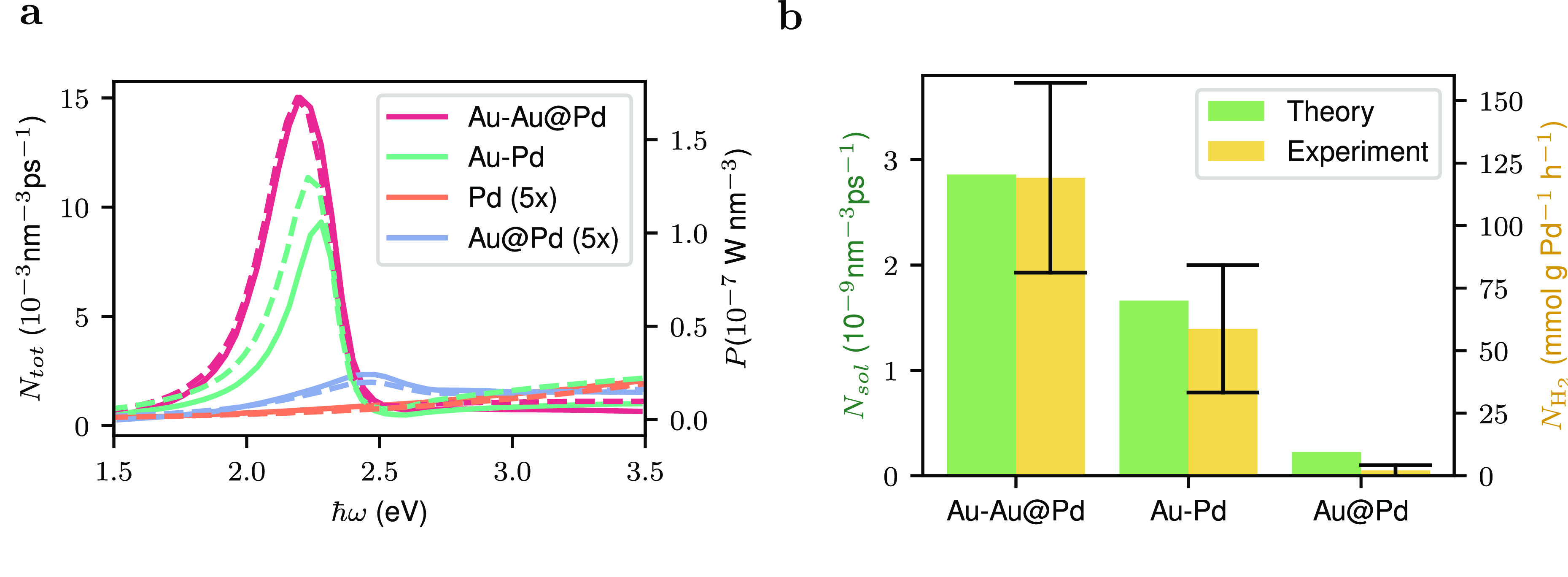
(a) Total hot carrier generation rate as a function of photon energy (averaged over all polarization vectors) for a spherical Pd nanoparticle, a Au@Pd core–shell nanoparticle, a Au–Pd reactor, and a Au–Au@Pd reactor. The power (per unit volume) absorbed by the Pd subsystem is shown in dashed lines. Note that the curves for the Pd nanoparticles and the Au@Pd have been multiplied by a factor of 5 to improve visibility. (b) Comparison of the measured difference in H_2_ production upon illumination by a solar simulator^26^ and in the dark to the calculated number of hot holes excited by solar illumination.

We compare the total Pd hot-carrier generation rates of the four different systems (spherical Pd nanoparticle, Au@Pd core–shell nanoparticle, Au–Pd antenna–reactor system, and Au–Au@Pd antenna–reactor system) in Figure 7a. It can be seen that the two antenna–reactor systems produce significantly more hot carriers in the Pd than the other two systems. In particular, a dramatic increase in the generation rate is observed near the plasmon frequency of ∼2.2 eV. As discussed above, the large generation rates are a consequence of the gap plasmon mode, which gives rise to large electric field enhancements. In the antenna–reactor system with a core–shell satellite, the electric field is more strongly confined because of the presence of the Au inside the core–shell satellite and therefore this system gives rise to the largest hot-carrier generation rate overall.

In their experiments, Herran and co-workers measure the increase in H_2_ production from formic acid by bimetallic plasmonic catalysts upon illumination with a solar simulator (see Figure 2a of ref (26)). In Figure 7b, we show the measured difference in H_2_ production in the dark and upon illumination for a Au@Pd core–shell nanoparticle, a Au–Pd antenna–satellite system, and a Au–Au@Pd system. We compare this difference to the total number of hot holes *N*_sol_ generated by the solar spectrum *S*(ω) (using the Air Mass 1.5 spectrum) obtained via
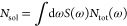
7

In excellent agreement with experiment, we find that the Au–Au@Pd antenna–satellite system is the best bimetallic plasmonic photocatalyst, while the Au@Pd system performs much worse. We note that the agreement between theory and experiment is somewhat worse for the Au@Pd system; this is likely a consequence of the different nanoparticle sizes used in the experiment and in our calculations and also of the neglect of charge transfer between core and shell in our model.

We note that we did not include hot-carrier relaxation effects into our calculations. Moreover, we did not include a detailed description of the interaction of the hot carriers with reactive molecular species. Extending our formalism for modeling hot-carrier generation rates to include these effects will be the topic of future work. Despite this, the correlation between our theory and the experimental results is remarkable, as shown in Figure 7b.

## Conclusion

We have studied hot-carrier generation in Au–Pd nanoarchitectures using an atomistic quantum-mechanical modeling approach. We have found that Au–Pd antenna-reactor systems exhibit hot-carrier generation rates significantly higher than those of core–shell nanoparticles. This is caused by the large electric field enhancements due to the localized plasmon mode associated with the gap between the antenna and the satellite nanoparticles. In particular, the largest overall hot-carrier generation rate is found for an antenna–reactor system, in which the satellite is a core–shell nanoparticle. For the antenna–reactor systems, we also studied the dependence of the hot-carrier generation rates on the size of the gap, the radius of the antenna nanoparticle, and the light polarization direction. We find that the largest rates are found when the electric field is parallel to the axis connecting the centers of the antenna and satellite nanoparticles. Also, small gaps and large antenna sizes favor hot-carrier generation. Comparing our calculated hot-carrier generation rates for the different Au–Pd photocatalysts to experimentally measured H_2_ production rates, we find a strong correlation between theory and experiment. The insights from our work can guide the development of highly efficient heterogeneous hot-carrier nanodevices for energy conversion applications.
